# Supplementary material for: Tumor necrosis factor mediates USE1-independent FAT10ylation under inflammatory conditions
Source: Life Sci Alliance. 2023 Aug 21;6(11):e202301985. doi: 10.26508/lsa.202301985 (PMC10442930; doi:10.26508/lsa.202301985)

Fig. 1A

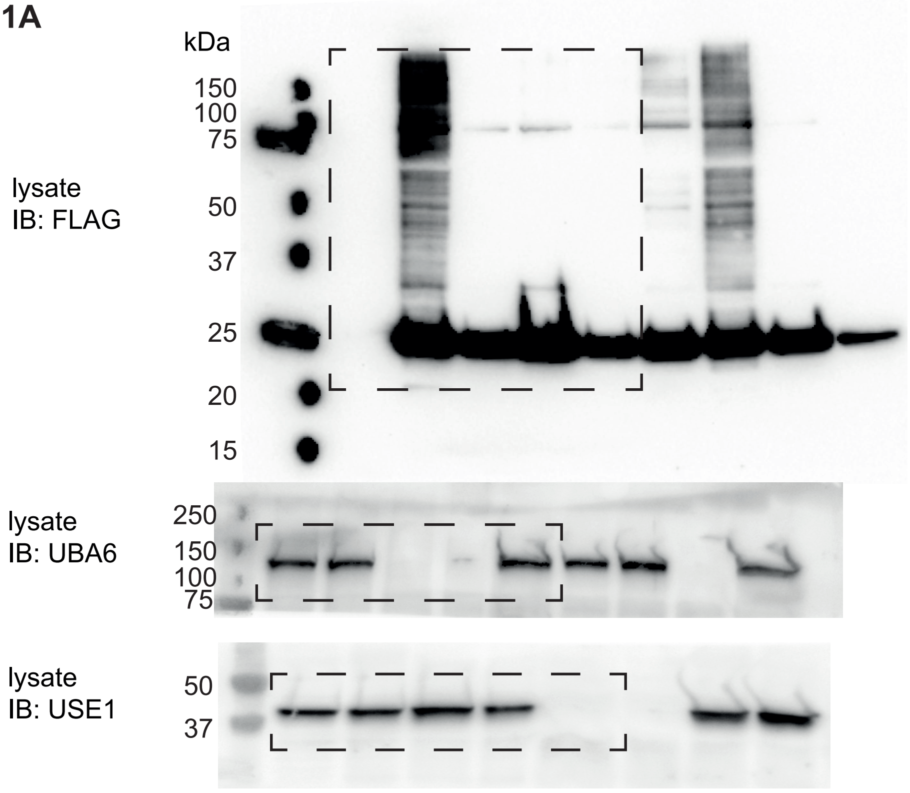

Fig. 1B

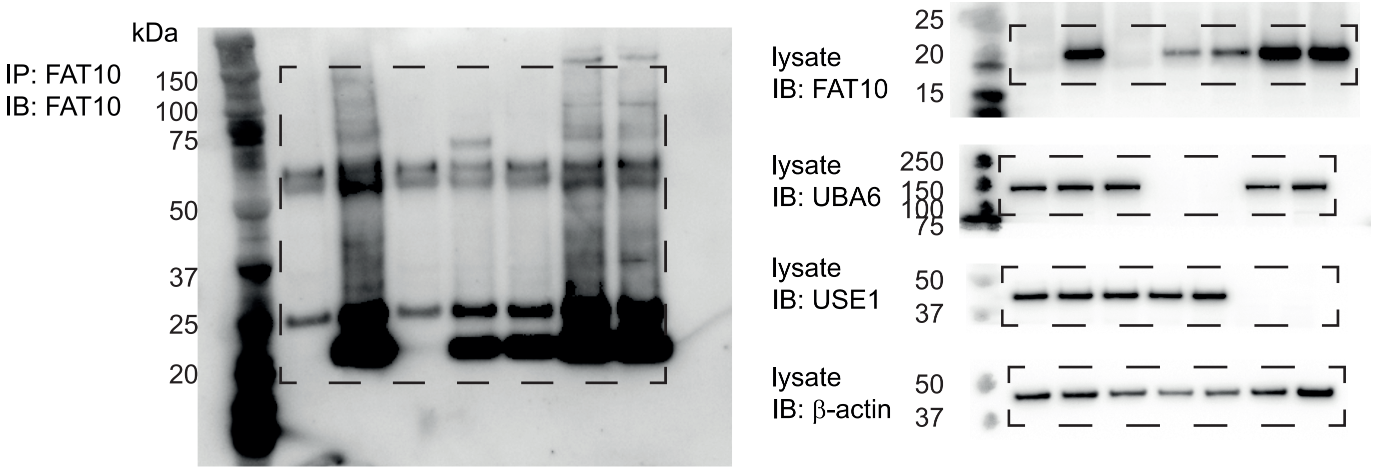

Fig. 1C

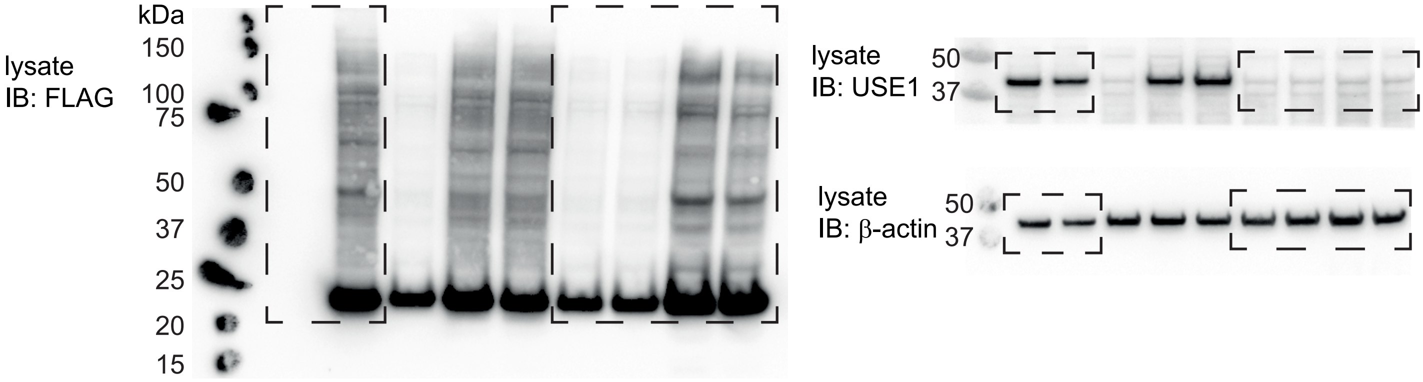

Fig. 1D

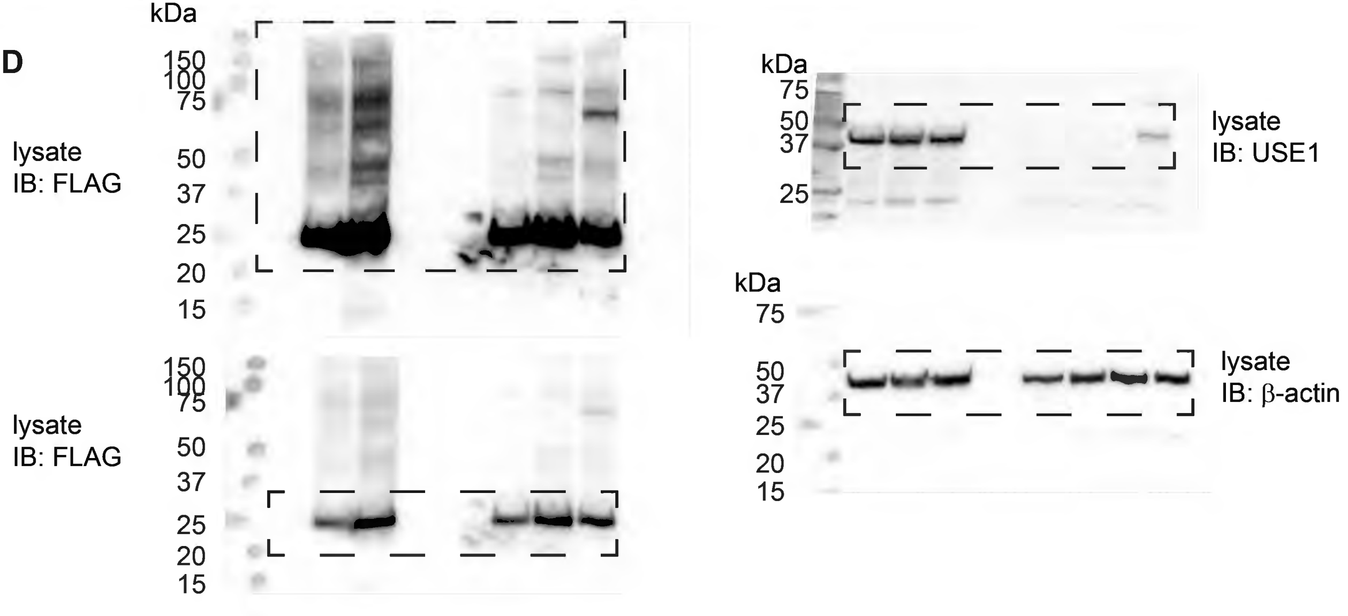
kDa
150
100
75
50
37
25
20
15
lysate
IB: FLAG
150
100
75
50
37
25
20
15
lysate
IB: FLAG
kDa
75
50
37
25
lysate
IB: USE1
kDa
75
50
37
25
20
15
lysate
IB: β-actin

Fig. 1E

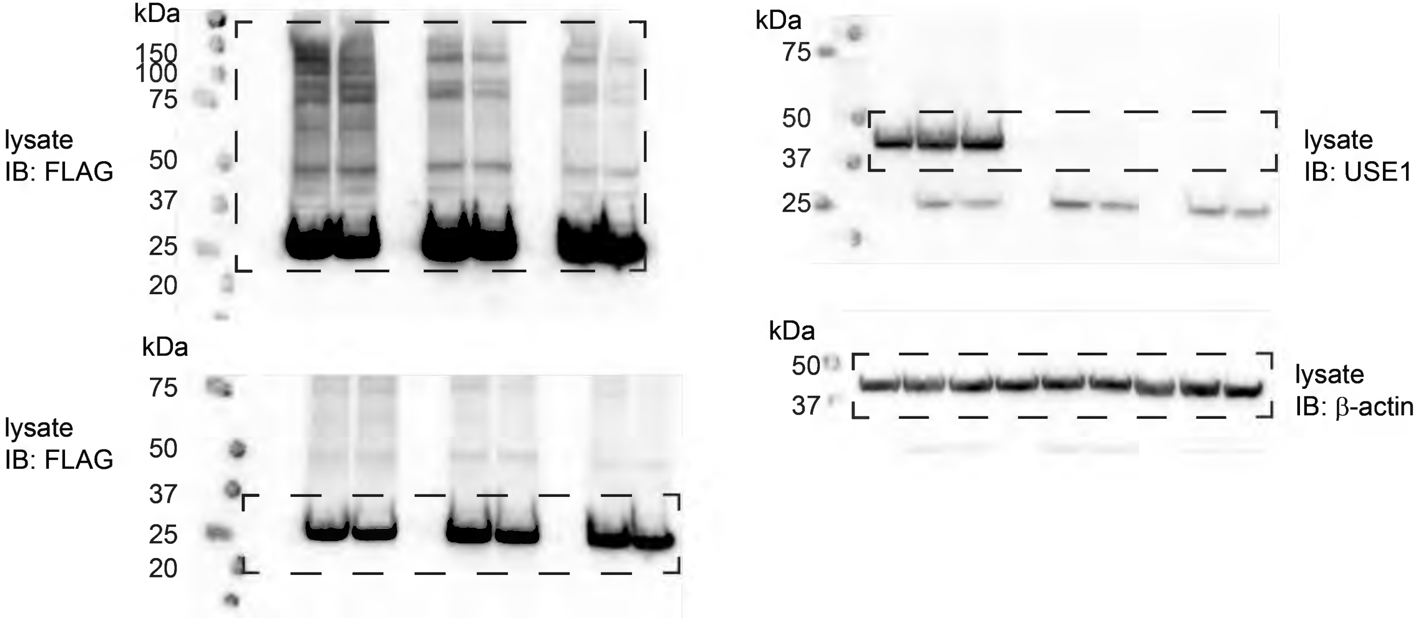
kDa
150
100
75
50
37
25
20
lysate
IB: FLAG
kDa
75
50
37
25
20
lysate
IB: FLAG
kDa
75
50
37
25
lysate
IB: USE1
kDa
50
37
lysate
IB: β-actin

Fig. 3A

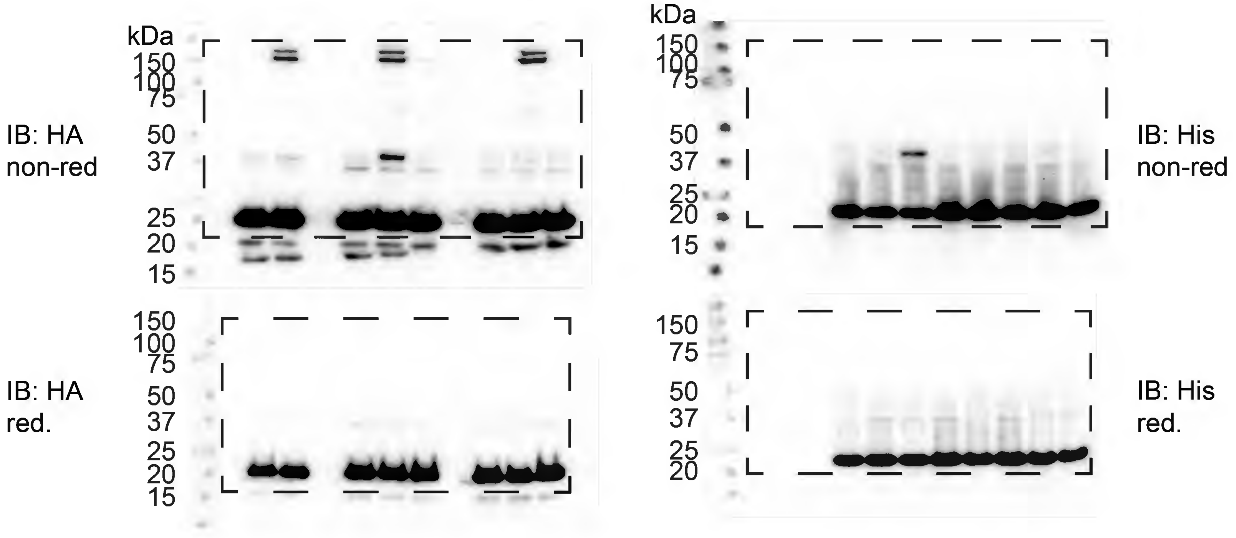

Fig. 3B

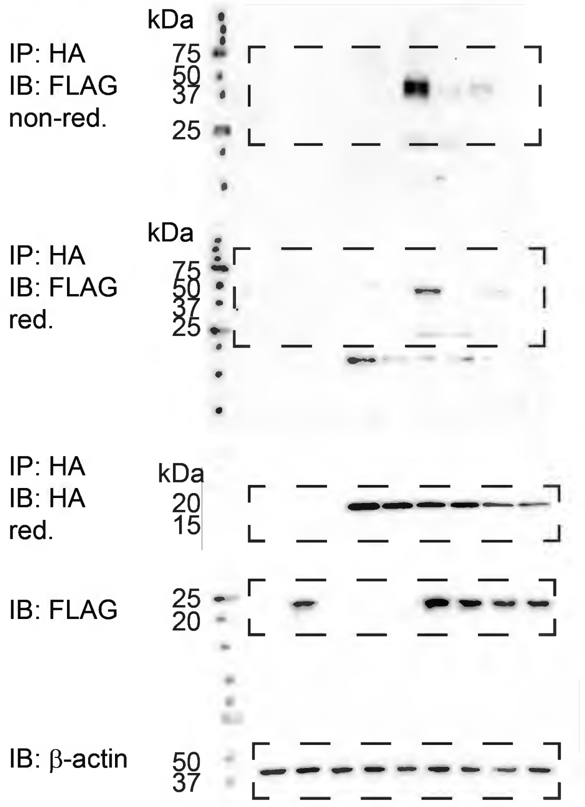

Fig. 3C

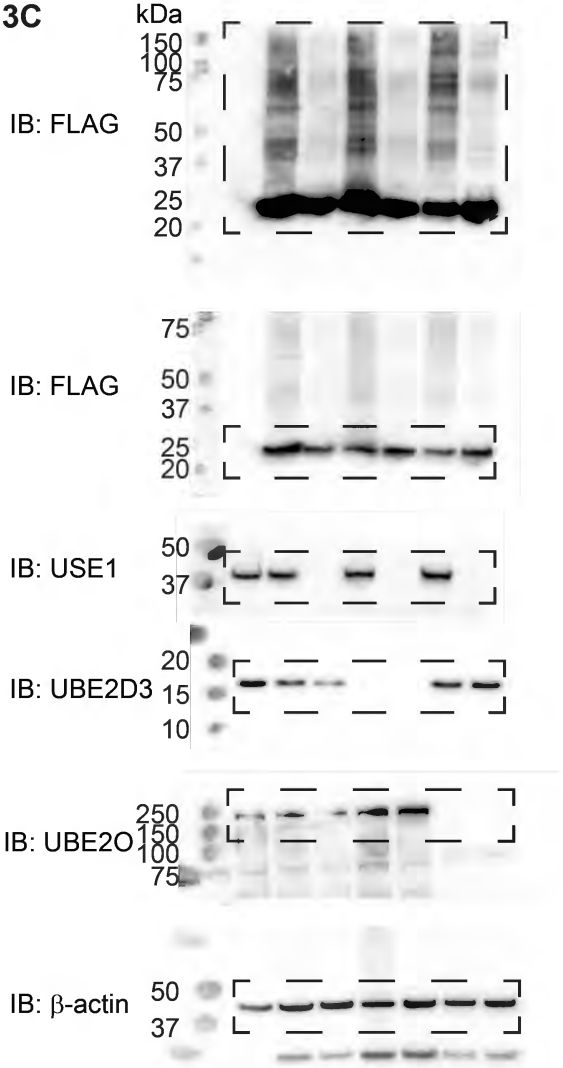

Fig. 3D

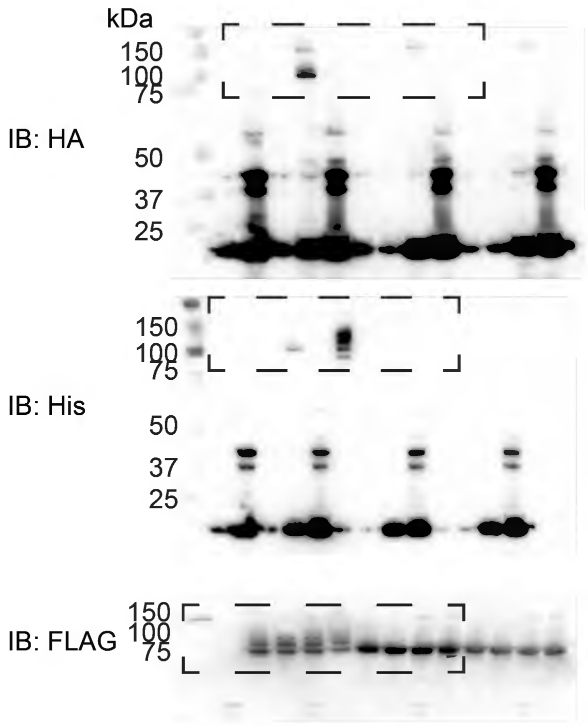
kDa
150
100
75
IB: HA
50
37
25
150
100
75
IB: His
50
37
25
IB: FLAG
150
100
75

Fig. 3E

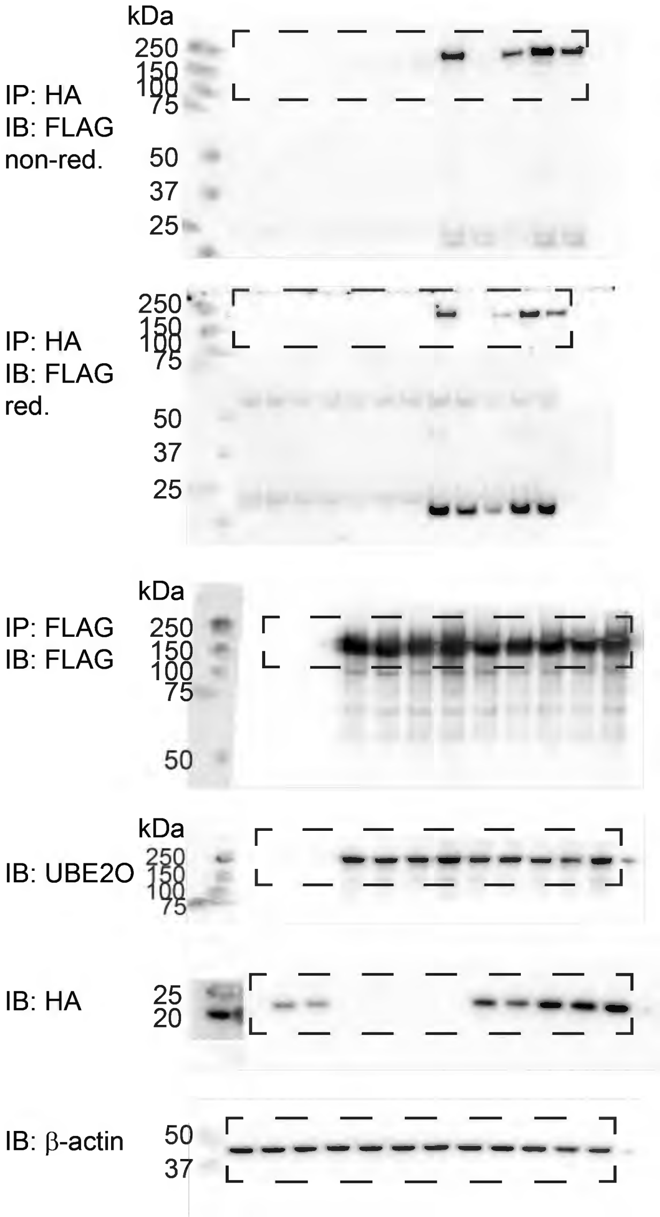
kDa
250
150
100
75
IP: HA
IB: FLAG
non-red.
50
37
25
250
150
100
75
IP: HA
IB: FLAG
red.
50
37
25
kDa
250
IP: FLAG
IB: FLAG
150
100
75
50
kDa
IB: UBE2O
250
150
100
75
IB: HA
25
20
IB: β-actin
50
37

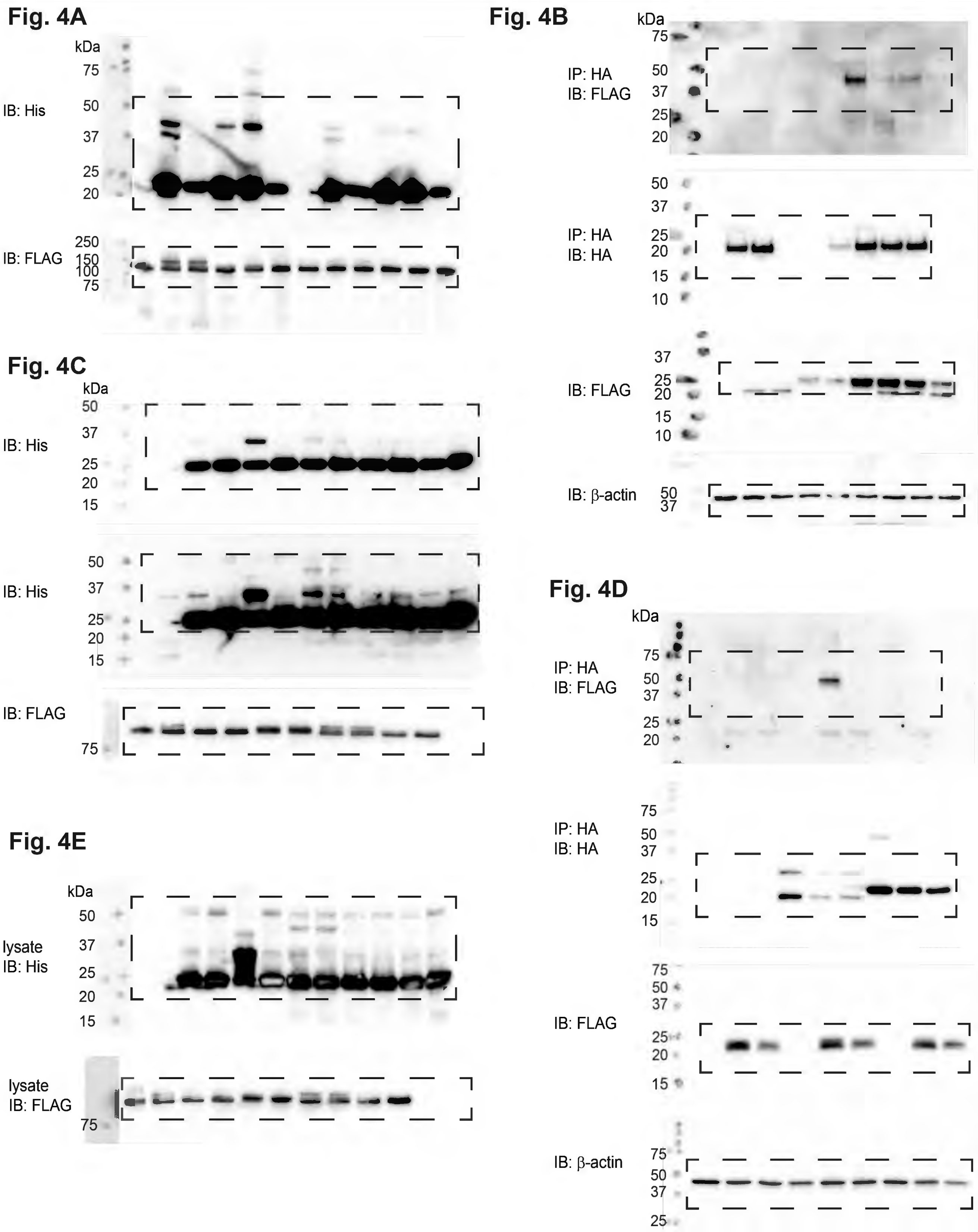

Fig. 4A
kDa
75
50
37
25
20
IB: His
250
150
100
75
IB: FLAG
Fig. 4B
kDa
75
50
37
25
20
IP: HA
IB: FLAG
50
37
25
20
15
10
IP: HA
IB: HA
37
25
20
15
10
IB: FLAG
IB: β-actin
50
37
Fig. 4C
kDa
50
37
25
20
15
IB: His
50
37
25
20
15
IB: His
IB: FLAG
75
Fig. 4D
kDa
75
50
37
25
20
IP: HA
IB: FLAG
75
50
37
25
20
15
IP: HA
IB: HA
75
50
37
25
20
15
IB: FLAG
75
50
37
25
IB: β-actin
Fig. 4E
kDa
50
37
25
20
15
lysate
IB: His
lysate
IB: FLAG
75

Fig. 4F

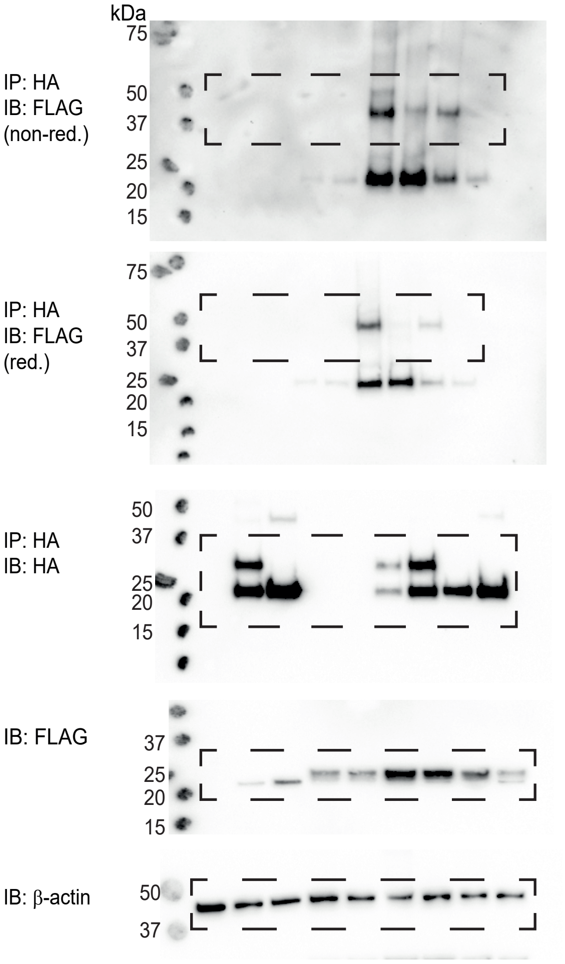

Fig. 4G

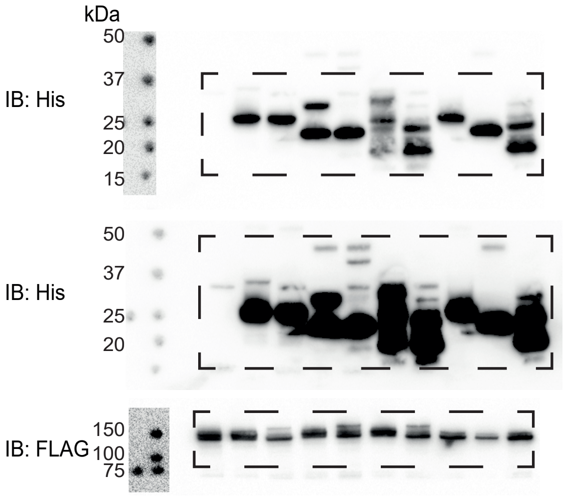

Fig. 4H

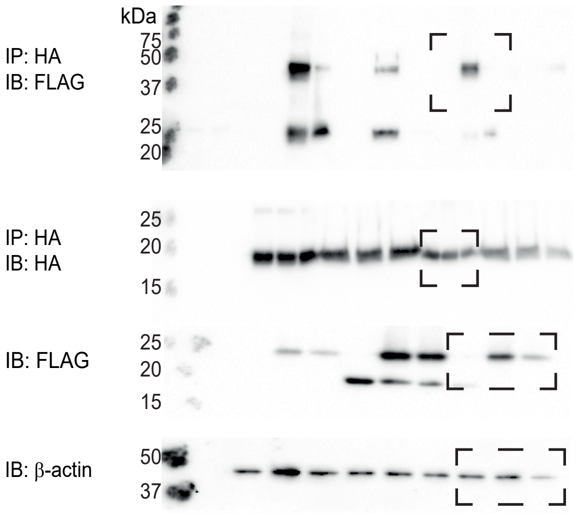

Fig. 5A

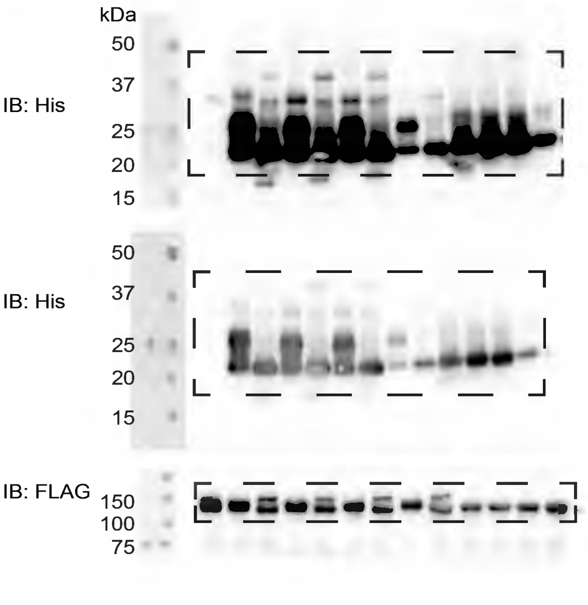

Fig. 5B

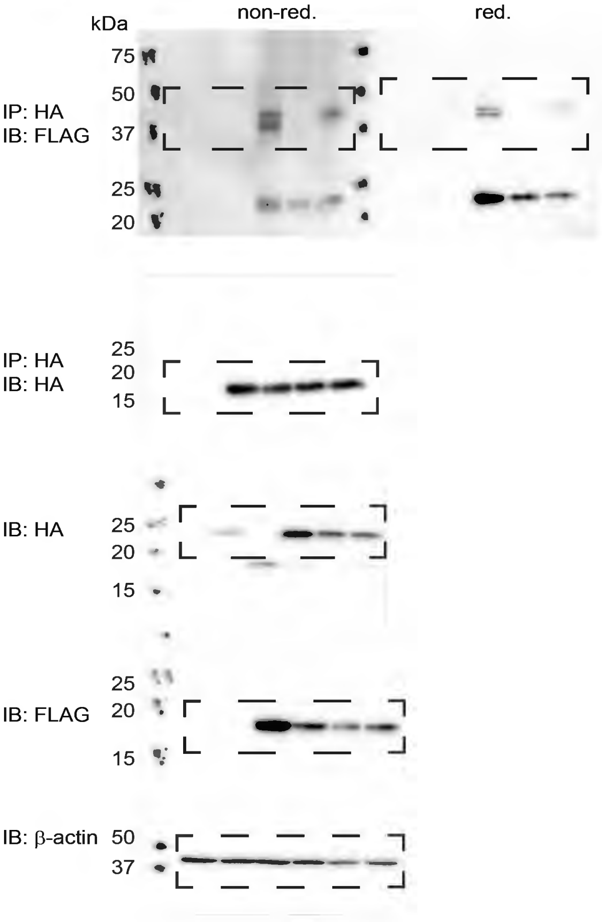

Fig. 5C

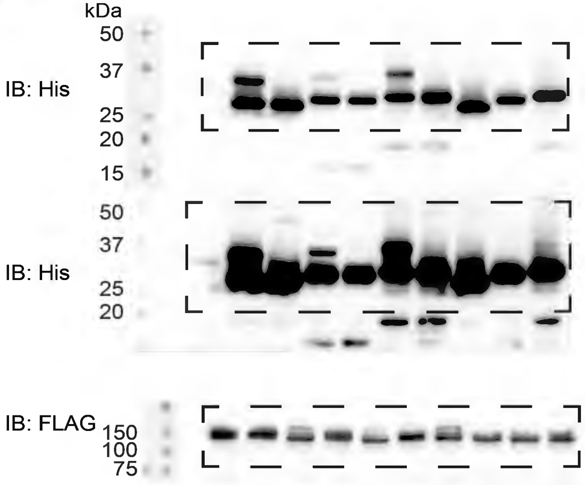

Fig. 6A

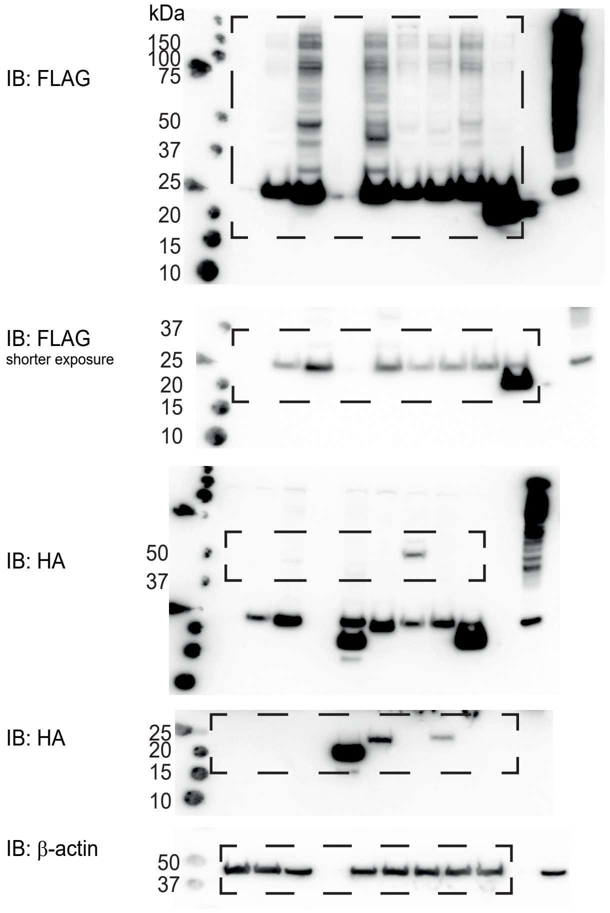

Fig. 6B

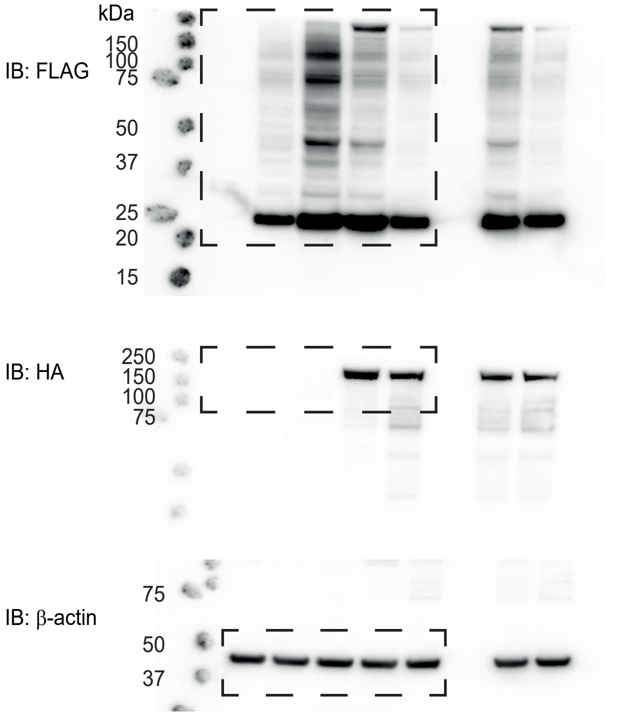

Fig. 6C

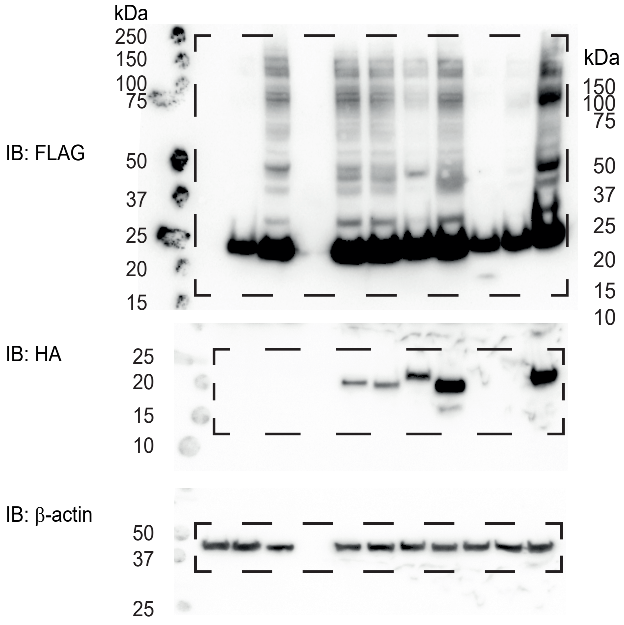

Fig. 6D

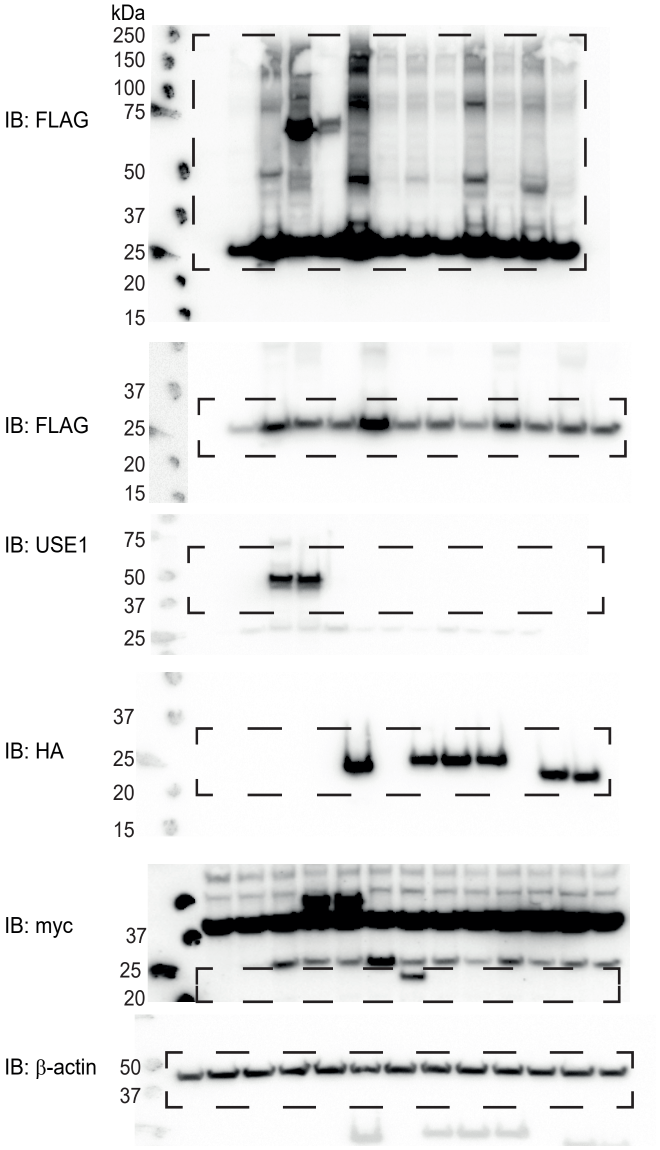

Fig. 7A

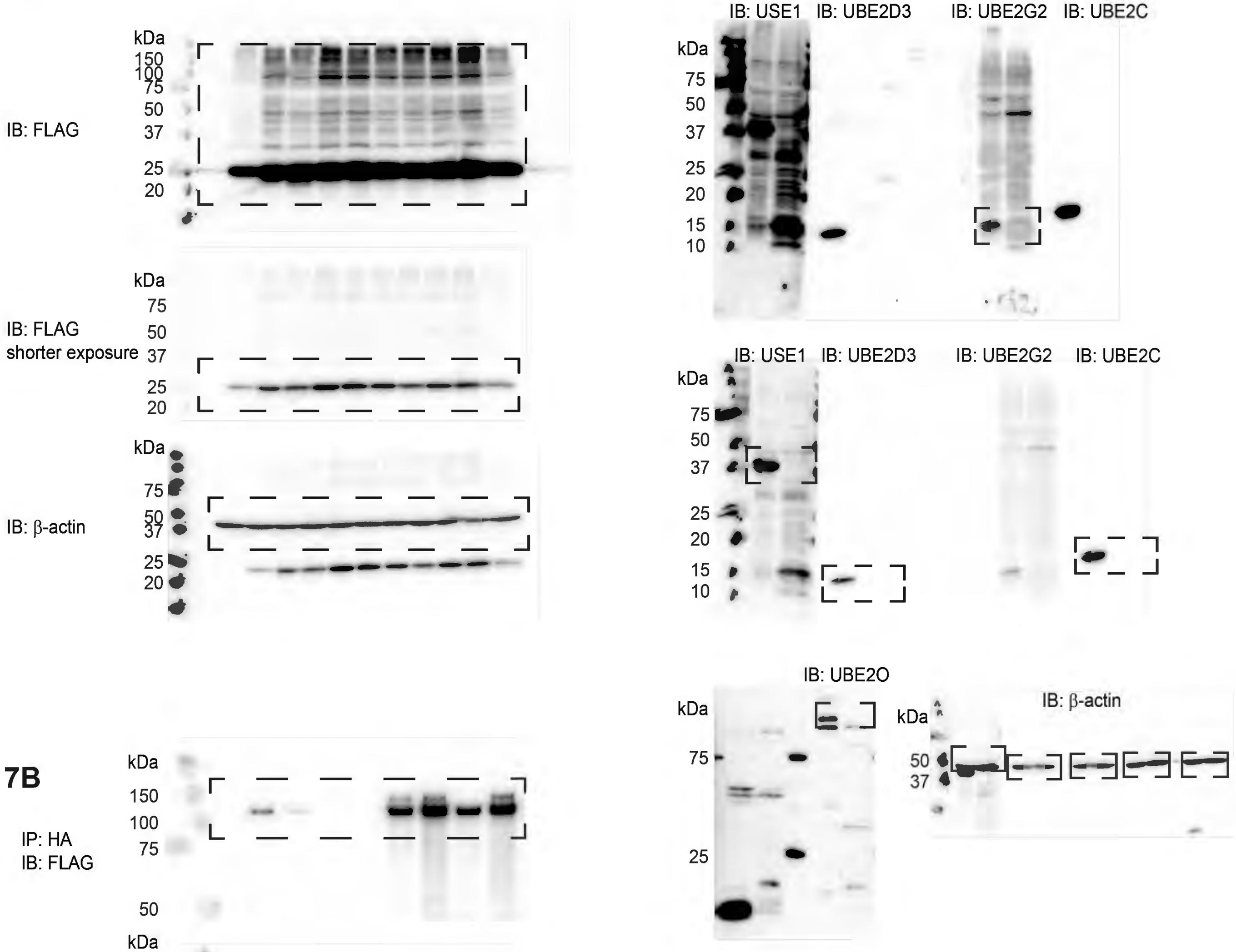
kDa
150
100
75
50
37
25
20
IB: FLAG
kDa
75
50
37
25
20
IB: FLAG
shorter exposure
kDa
75
50
37
25
20
IB: β-actin
IB: USE1
IB: UBE2D3
IB: UBE2G2
IB: UBE2C
kDa
75
50
37
25
20
15
10
IB: USE1
IB: UBE2D3
IB: UBE2G2
IB: UBE2C
kDa
75
50
37
25
20
15
10
IB: UBE2O
kDa
75
25
IB: β-actin
kDa
50
37

Fig. 7B

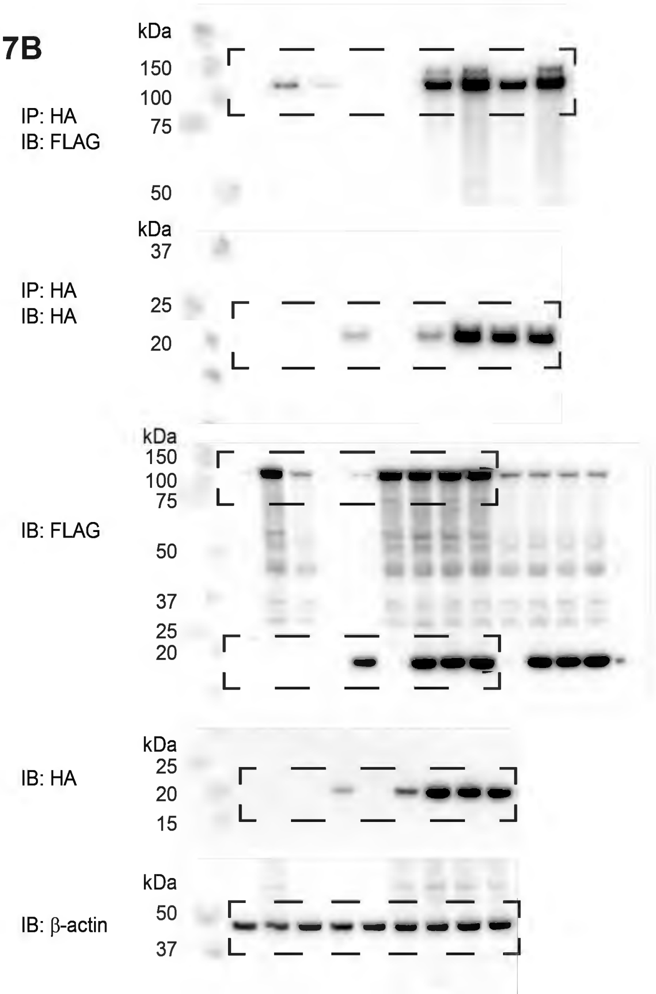
kDa
150
100
75
50
IP: HA
IB: FLAG
kDa
37
25
20
IP: HA
IB: HA
kDa
150
100
75
50
37
25
20
IB: FLAG
kDa
25
20
15
IB: HA
kDa
50
37
IB: β-actin

Fig. 7C

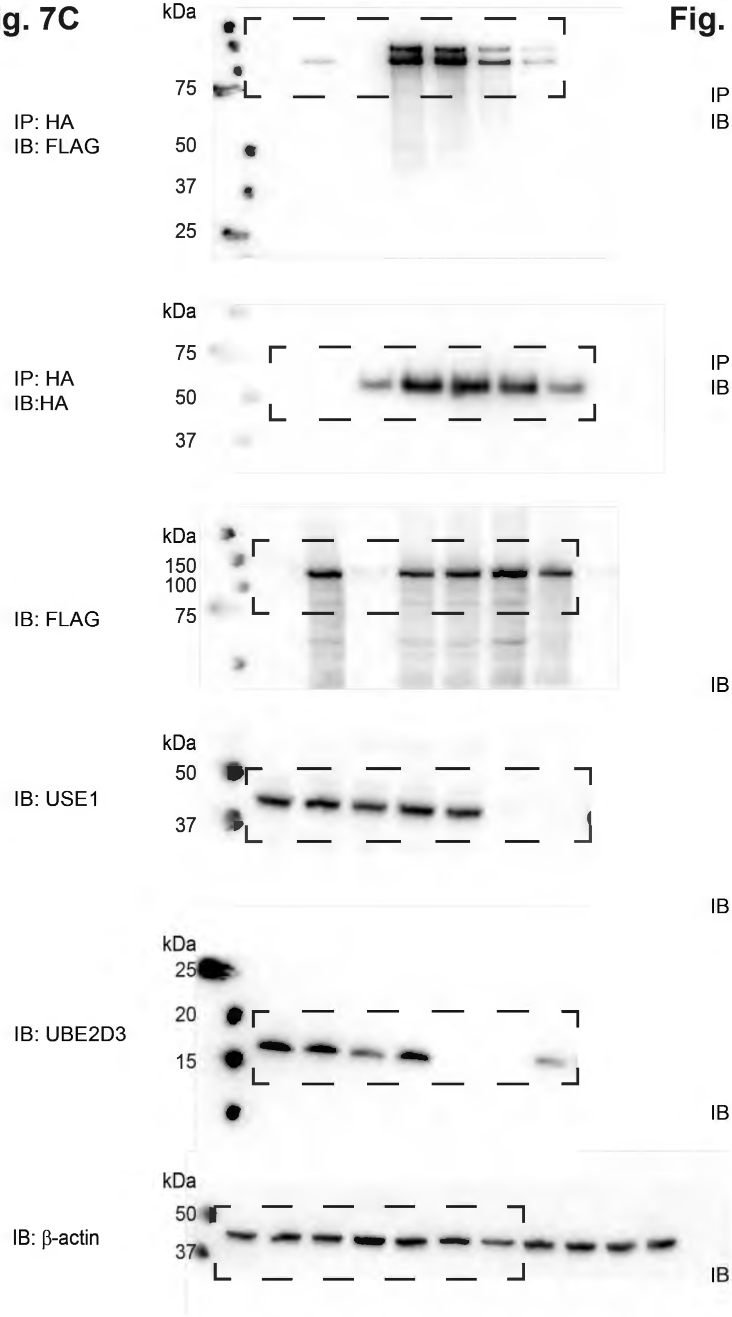
IP: HA
IB: FLAG
kDa
75
50
37
25
IP: HA
IB:HA
kDa
75
50
37
IB: FLAG
kDa
150
100
75
IB: USE1
kDa
50
37
IB: UBE2D3
kDa
25
20
15
IB: β-actin
kDa
50
37

Fig. 7D

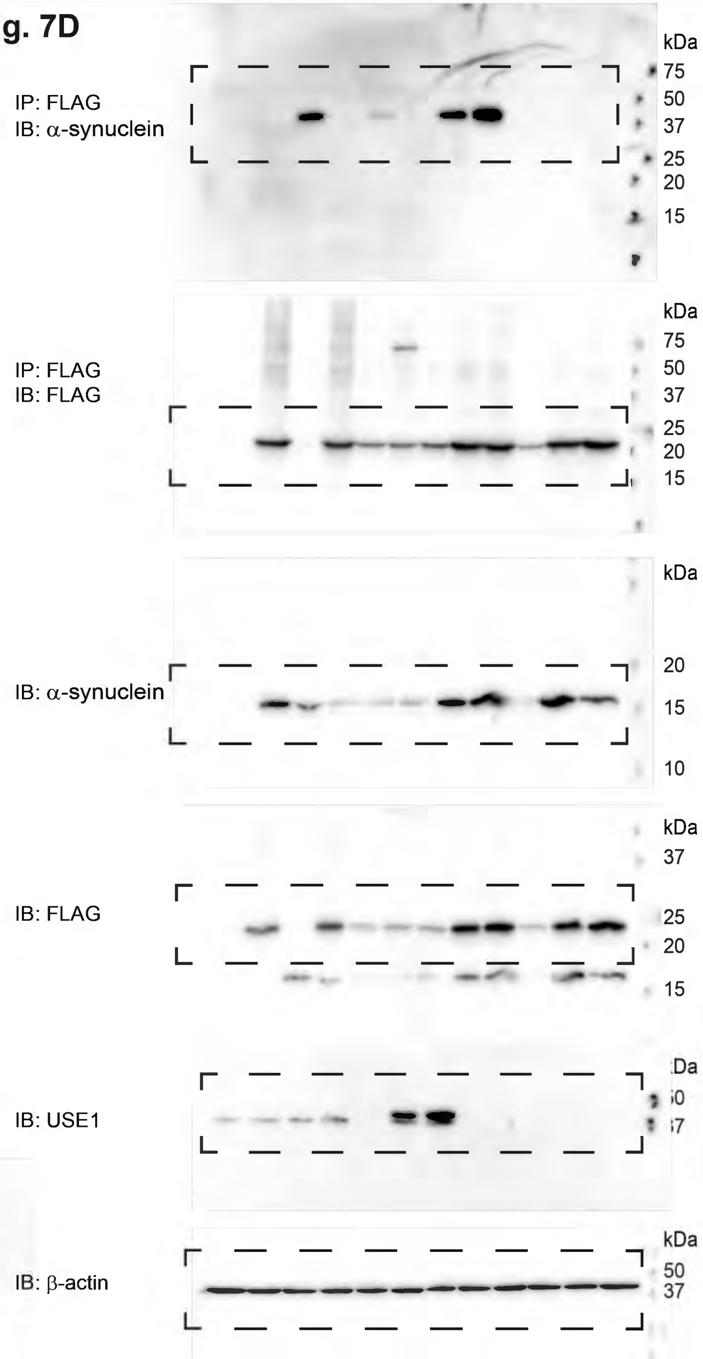
IP: FLAG
IB: α-synuclein
kDa
75
50
37
25
20
15
IP: FLAG
IB: FLAG
kDa
75
50
37
25
20
15
IB: α-synuclein
kDa
20
15
10
IB: FLAG
kDa
37
25
20
15
IB: USE1
IB: β-actin
kDa
50
37

Fig. 7E

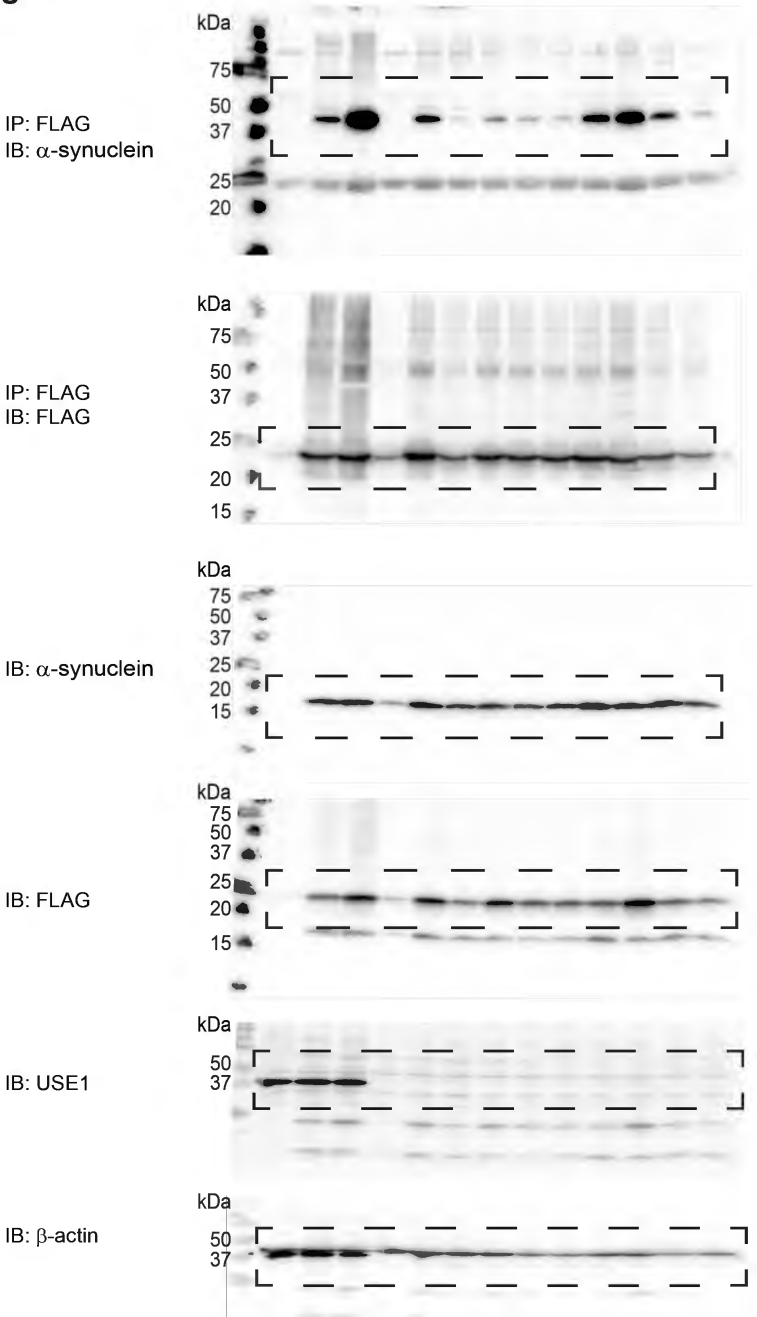
IP: FLAG
IB: α-synuclein
kDa
75
50
37
25
20
IP: FLAG
IB: FLAG
kDa
75
50
37
25
20
15
IB: α-synuclein
kDa
75
50
37
25
20
15
IB: FLAG
kDa
75
50
37
25
20
15
IB: USE1
kDa
50
37
IB: β-actin
kDa
50
37

Fig S1A

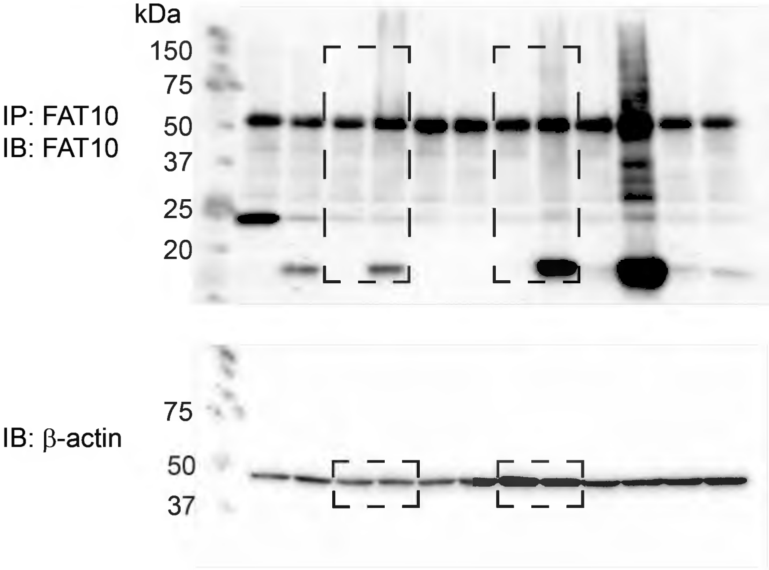

Fig S1B

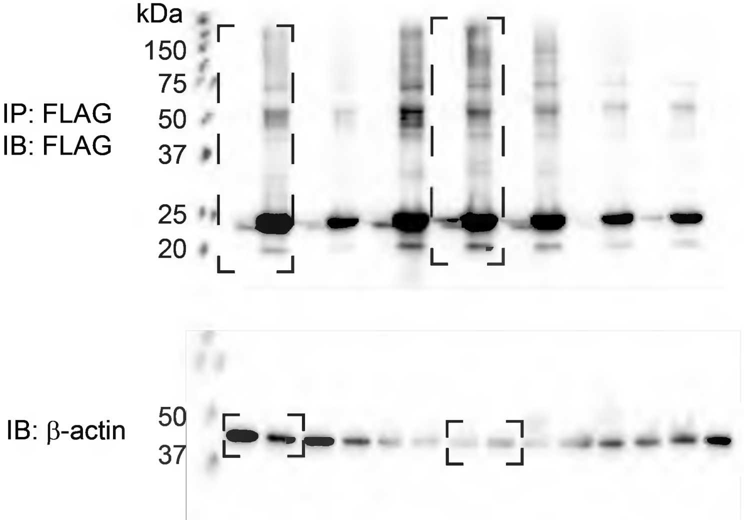

Fig S2A

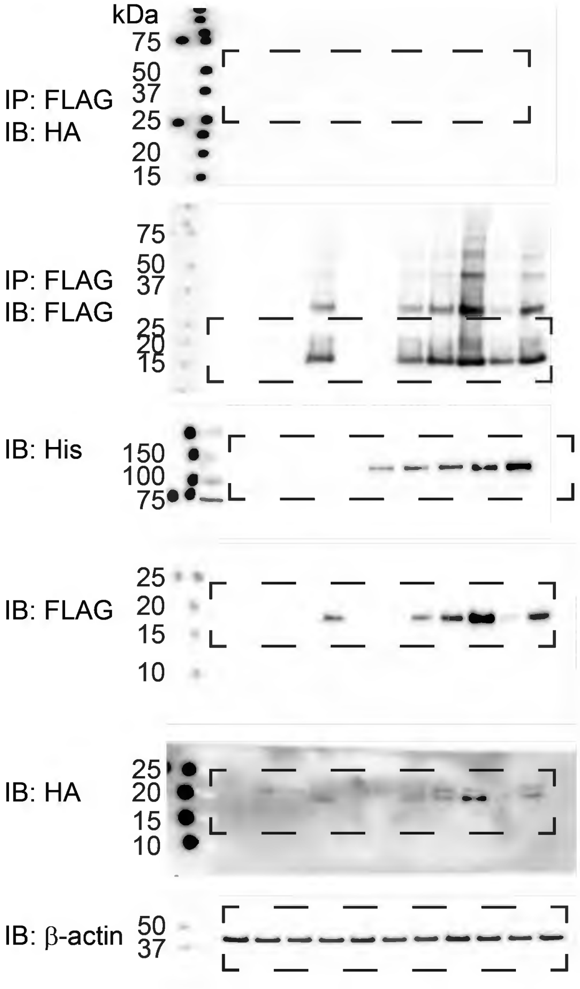

Fig S2B

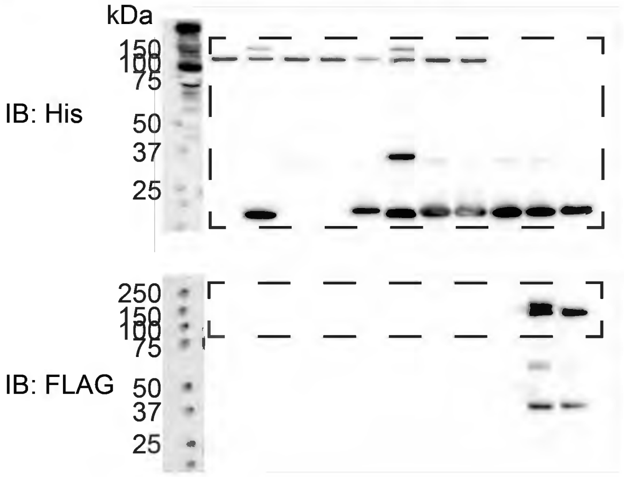

Fig S2C

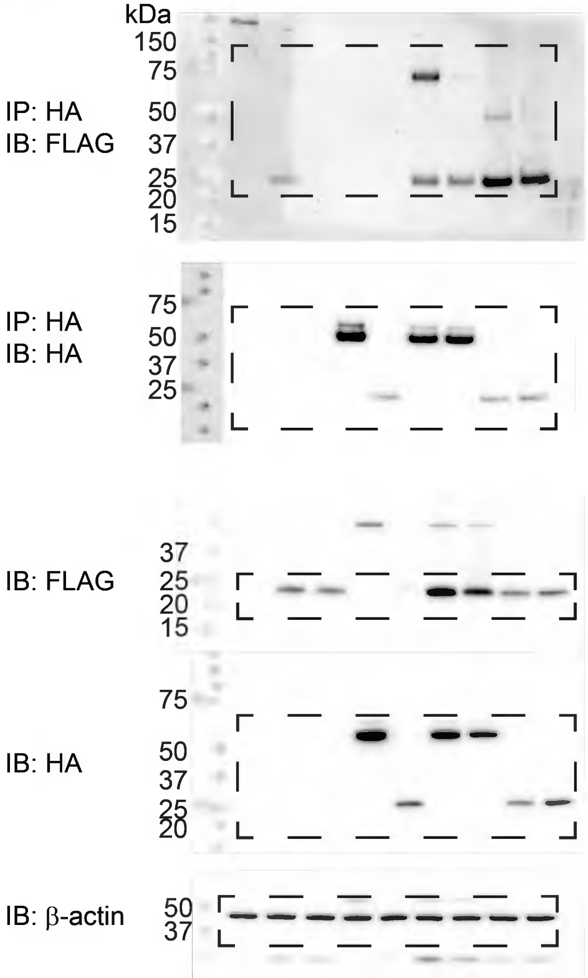

Fig S2D

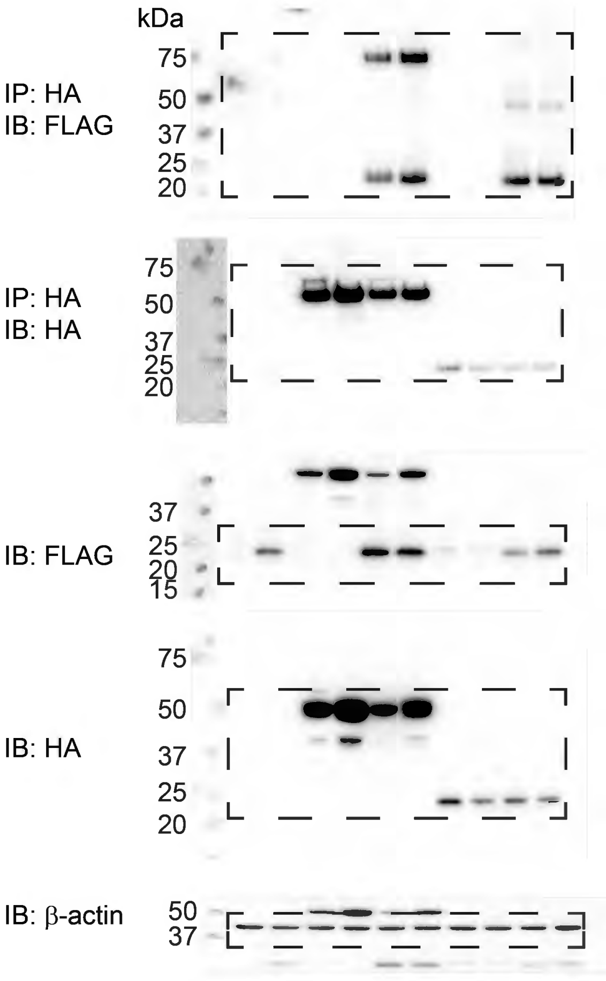

Fig S3

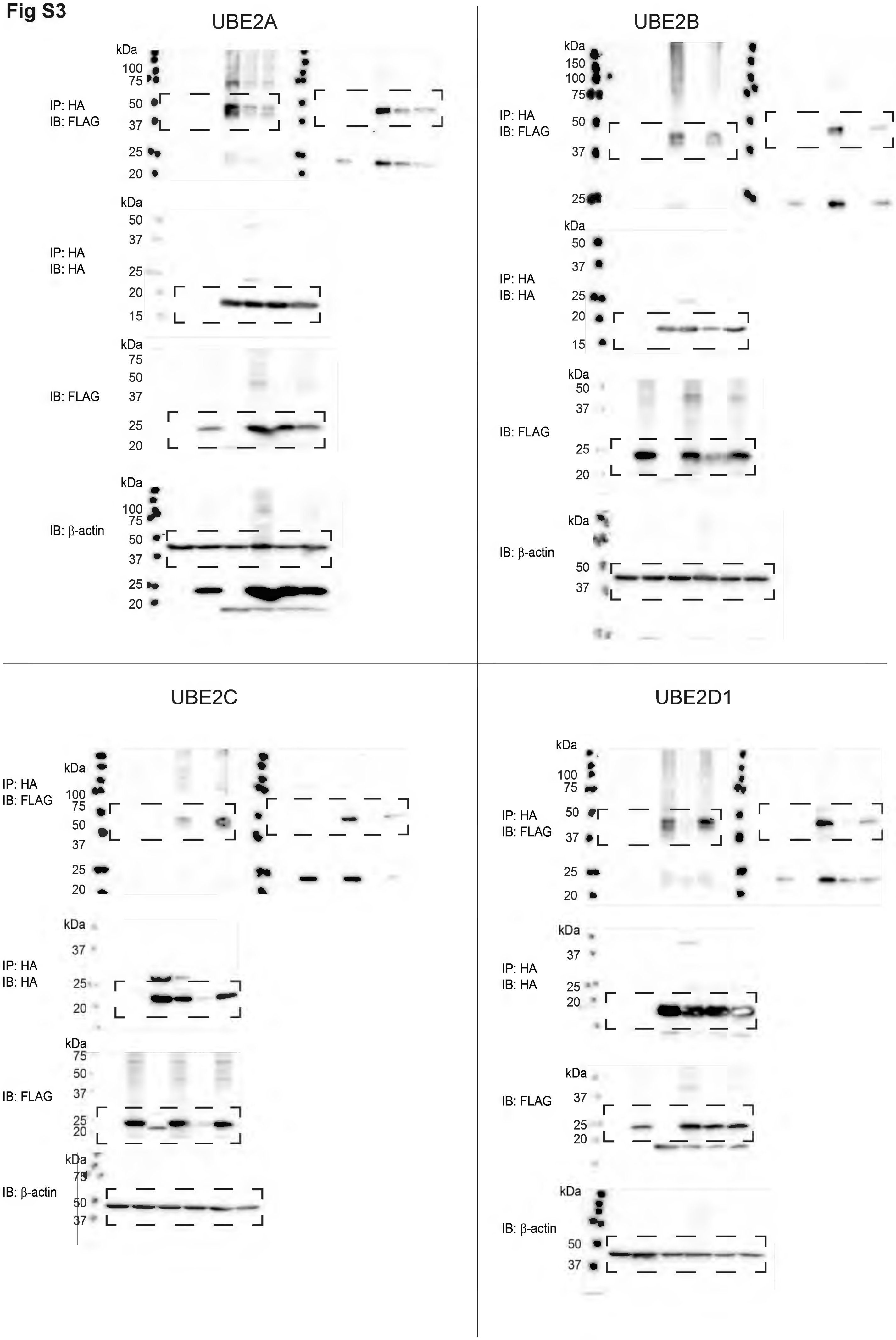
UBE2A
kDa
100
75
50
37
25
20
IP: HA
IB: FLAG
kDa
50
37
25
20
15
IP: HA
IB: HA
kDa
75
50
37
25
20
IB: FLAG
kDa
100
75
50
37
25
20
IB: β-actin
UBE2B
kDa
150
100
75
50
37
25
IP: HA
IB: FLAG
kDa
50
37
25
20
15
IP: HA
IB: HA
kDa
50
37
25
20
IB: FLAG
kDa
50
37
IB: β-actin
UBE2C
kDa
100
75
50
37
25
20
IP: HA
IB: FLAG
kDa
37
25
20
IP: HA
IB: HA
kDa
75
50
37
25
20
IB: FLAG
kDa
75
50
37
IB: β-actin
UBE2D1
kDa
100
75
50
37
25
20
IP: HA
IB: FLAG
kDa
37
25
20
IP: HA
IB: HA
kDa
37
25
20
IB: FLAG
kDa
50
37
IB: β-actin

Fig S3

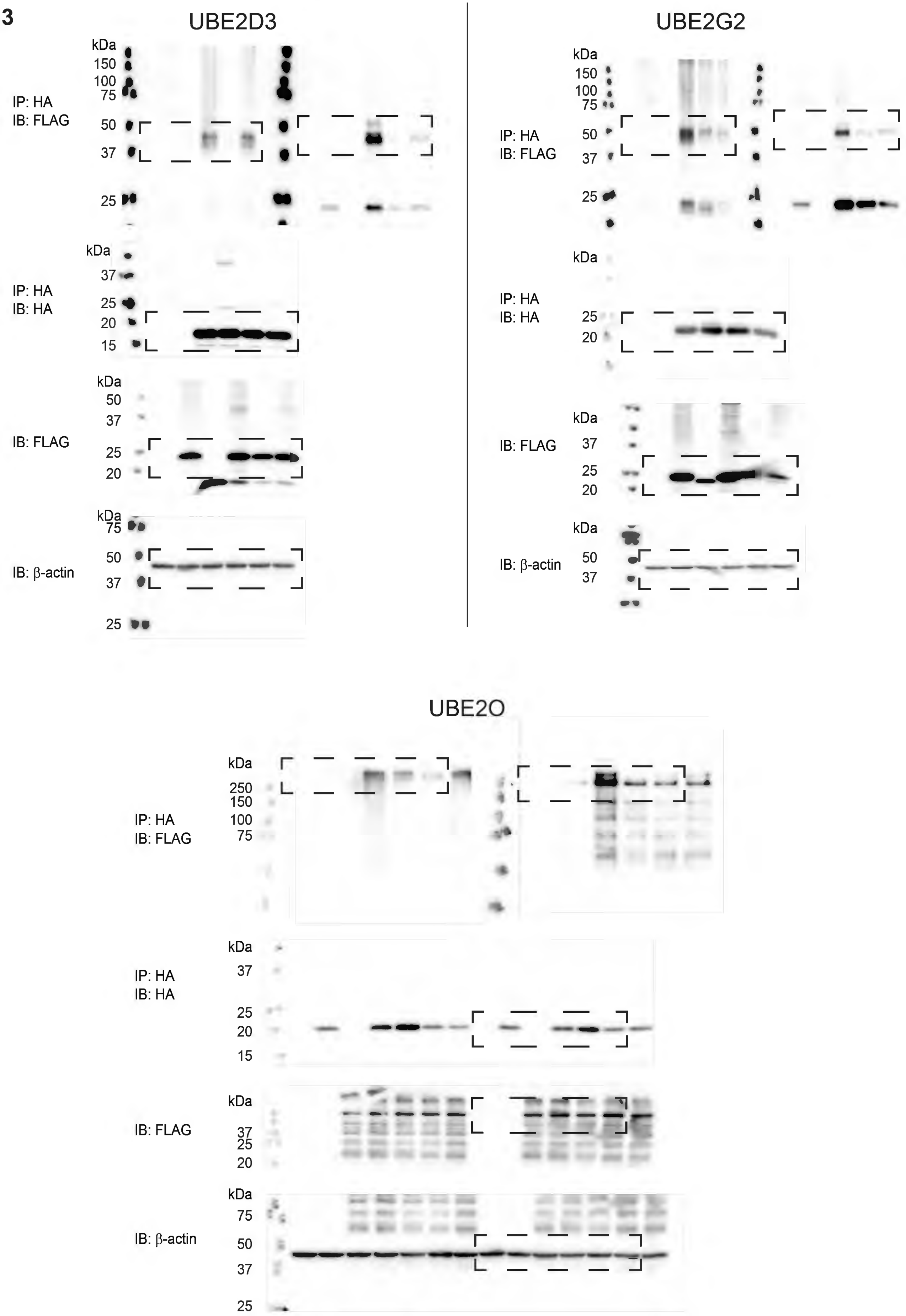
UBE2D3
kDa
150
100
75
50
37
25
IP: HA
IB: FLAG
kDa
37
25
20
15
IP: HA
IB: HA
kDa
50
37
25
20
IB: FLAG
kDa
75
50
37
25
IB: β-actin
UBE2G2
kDa
150
100
75
50
37
25
IP: HA
IB: FLAG
kDa
25
20
IP: HA
IB: HA
kDa
37
25
20
IB: FLAG
kDa
50
37
IB: β-actin
UBE2O
kDa
250
150
100
75
IP: HA
IB: FLAG
kDa
37
25
20
15
IP: HA
IB: HA
kDa
37
25
20
IB: FLAG
kDa
75
50
37
25
IB: β-actin

Fig S4A

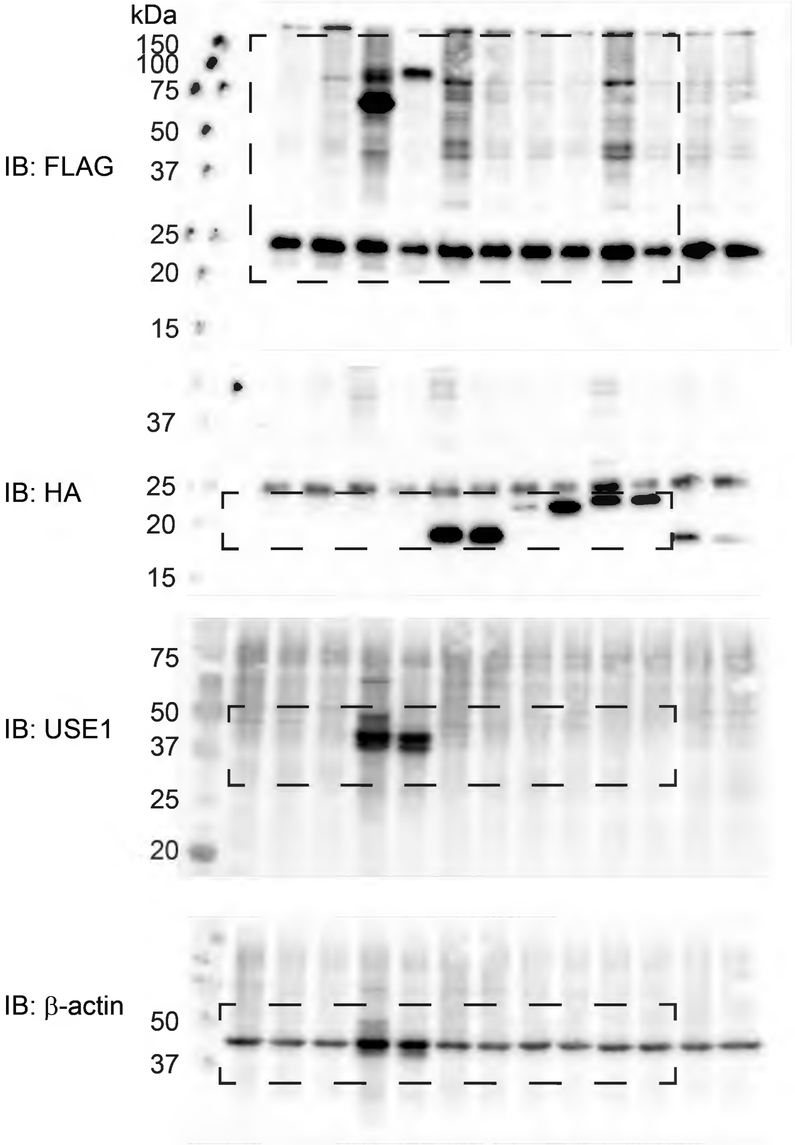

Fig S4B

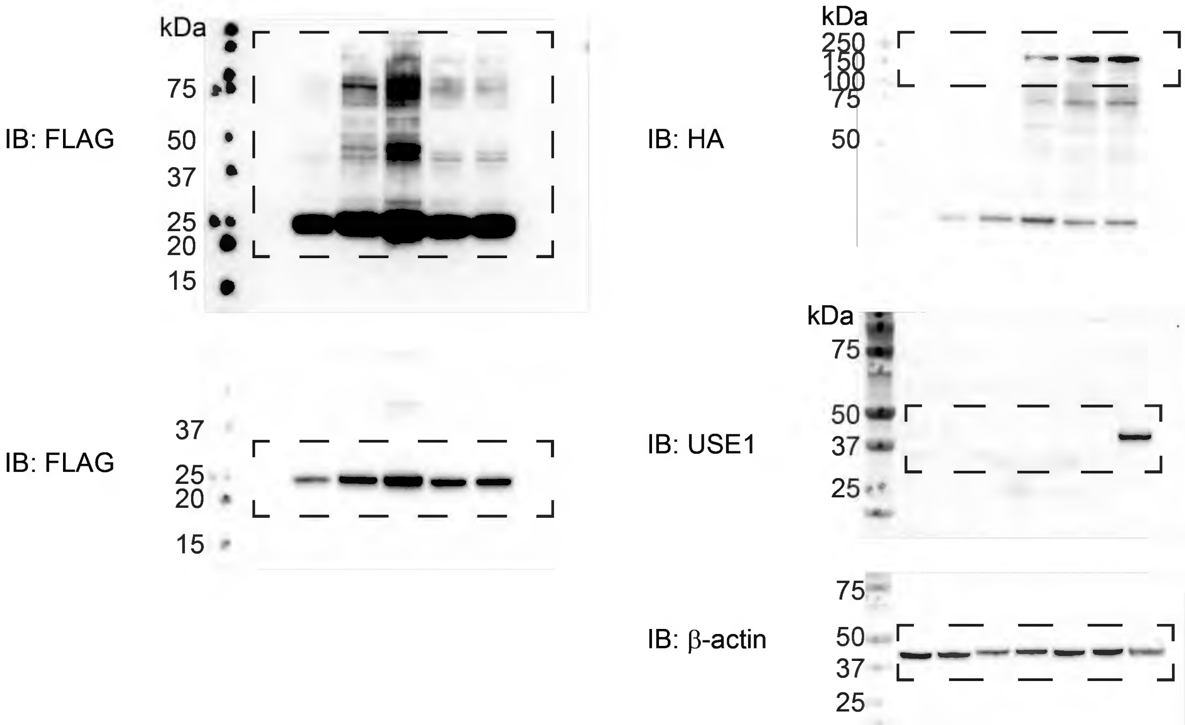

Fig S5A

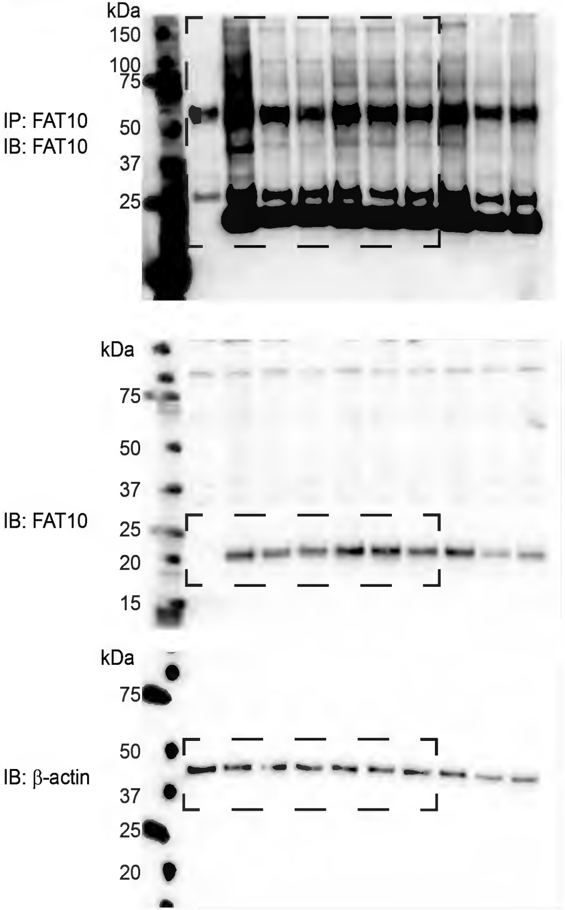

Fig S5B

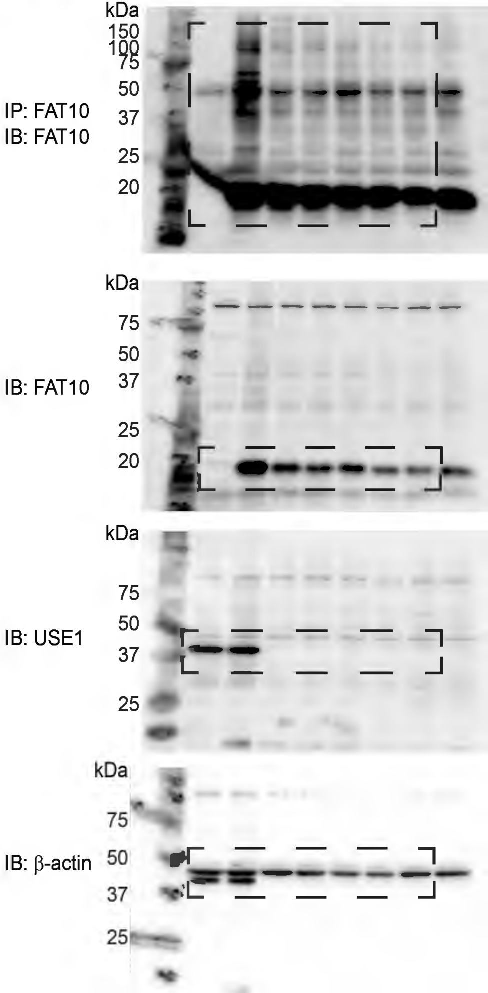

Supplement: Supplementary file 1 [file LSA-2023-01985_SdataF1_F3_F4_F5_F6_F7_FS1_FS2_FS3_FS4_FS5.pdf]
