# Supplementary material for: Tumor necrosis factor mediates USE1-independent FAT10ylation under inflammatory conditions
Source: Life Sci Alliance. 2023 Aug 21;6(11):e202301985. doi: 10.26508/lsa.202301985 (PMC10442930; doi:10.26508/lsa.202301985)
Supplement: Supplementary file 2 [file LSA-2023-01985_TableS1.docx]

**Table S1.**

| **Plasmid** | **Reference** | **Oligonucleotides used for PCR cloning** | **Restrictions enzymes used for cloning** |
| --- | --- | --- | --- |
| pcDNA3.1-His/-A-USE1 | ([Aichem et al, 2010](#_ENREF_1)) | **-** | - |
| pcDNA3.1-His/-A-USE1-C188A | ([Aichem et al, 2010](#_ENREF_1)) | **-** | - |
| pCMV-HA-UBE2A |  | AA-423 5’GGAAGATCTCTTCCACCCCGGCTCGGCGGCGC 3’  AA-424  5’CGGGGTACCTCAACAATCACGCCAGCTTTGTTCTAC3’ | BglII, KpnI |
| pCMV-HA-UBE2B |  | AA-427  5’ GGAAGATCTCTTCGACCCCGGCCCGGAGGAG 3’  AA-428  5’CGGGGTACCTTATGAATCATTCCAGCTTTGTTCAAC 3’ | BglII, KpnI |
| pCMV-HA-UBE2C |  | AA-382 5’GGCGAATTCGGGCTTCCCAAAACCGCGACCCAGC 3’  AA-383 5’GGTCACCAGCCAGGAGCCCTGACTCGAGCGG 3’ | EcoRI, XhoI |
| pCMV-HA-UBE2D1 |  | AA-425 5’ GGAAGATCTCTGCGCTGAAGAGGATTCAGAAAGAATTG 3’  AA-426  5’CGGGGTACCTTACATTGCATATTTCTGAGTCCATTC 3’ | BglII, KpnI |
| pcDNA3-FLAG-UBE2D2 (UbcH5b) | Gift from Martin Scheffner, University of Konstanz, Germany | - | - |
| pCMV-HA-UBE2D3 |  | AA-431  5’GGAAGATCTCTGCGCTGAAACGGATTAATAAGGAAC 3’  AA-432  5’CGGGGTACCTCACATGGCATACTTCTGAGTCC 3’ | BglII, KpnI |
| pcDNA3.1-HA-UBE2E1 | Addgene plasmid #118213  ([Pasupala et al, 2018](#_ENREF_4)) |  | - |
| pCMV-HA-UBE2F |  | AA-421  5’CGCGAATTCGGCTAACGCTAGCAAGTAAACTGAAGCG 3’  AA-422  5’CCGCTCGAGTCATCTGGCATAACGTTTGATGTAGTCATCC 3’ | EcoRI, XhoI |
| pCMV-HA-UBE2G2 |  | AA-429  5’GGAAGATCTCTGCGGGGACCGCGCTCAAGAGG 3’  AA-430  5’CGGGGTACCTCACAGTCCCAGAGACTTCTGG 3’ | BglII, KpnI |
| pcDNA3.1-FLAG-UBCH8 | ([Aichem et al, 2010](#_ENREF_1)) | - | - |
| pcDNA5FRT/TO-Strep-HA-UBE2Q2 | Addgene plasmid  #124668  ([Koerver et al, 2019](#_ENREF_3)) |  |  |
| pcDNA5FRT/TO-Strep-HA-UBE2QL1 | Addgene plasmid  #124665  ([Koerver et al, 2019](#_ENREF_3)) |  |  |
| pcDNA3.1-3xFLAG-TEV-UBE2O | Addgene plasmid  #105718  ([Yanagitani et al, 2017](#_ENREF_5)) |  |  |
| pcDNA3.1-3xFLAG-TEV-UBE2O-trunc  (nt2434-3879) |  | AA-450 5`CCGGAATTCGAGATAGAACCCGGGAGTTGAAAGAGGCCATCAAG 3`  AA-451 5`GCCGGAGTGCACAGAGGACAAGTAGTCTAGACTAG 3` | EcoRI, XbaI |
| pcDNA3.1-HA-TEV-UBE2O |  | PR6-125'-EcoRI-UBE2O  5'CCGGAATTCTCATGGCGGATCCCGCAGCCCCCACG 3'  PR6-133'-NotI-UBE2O  5'TTTTCCTTTTGCGGCCGCCTACTTGTCCTCTGTGCACTCCGGCATGCCTG 3' | EcoRI, NotI |
| pcDNA3.1-HA-TEV-UBE2O-C1040A |  | PR6-125'-EcoRI-UBE2O  5'CCGGAATTCTCATGGCGGATCCCGCAGCCCCCACG 3'  PR6-133'-NotI-UBE2O  5'TTTTCCTTTTGCGGCCGCCTACTTGTCCTCTGTGCACTCCGGCATGCCTG 3' | EcoRI, NotI |
| pcDNA3.1-HA-TEV-UBE2O C617/1040A |  | PR6-125'-EcoRI-UBE2O  5'CCGGAATTCTCATGGCGGATCCCGCAGCCCCCACG 3'  PR6-133'-NotI-UBE2O  5'TTTTCCTTTTGCGGCCGCCTACTTGTCCTCTGTGCACTCCGGCATGCCTG 3' | EcoRI, NotI |
