# Supplementary material for: Tumor necrosis factor mediates USE1-independent FAT10ylation under inflammatory conditions
Source: Life Sci Alliance. 2023 Aug 21;6(11):e202301985. doi: 10.26508/lsa.202301985 (PMC10442930; doi:10.26508/lsa.202301985)
Supplement: Supplementary file 3 [file LSA-2023-01985_TableS2.docx]

**Table S2.**

| **Plasmid** | **Active-site cysteine** | **Oligonucleotides used for site-directed mutagenesis of active-site cysteine** |
| --- | --- | --- |
| pcDNA3.1-His/-A-USE1-C188A | Cys188 | AA-103 5’CTACCGCAATGGGAAAGTCGCCTTGAGTATTCTAGGTACATGG 3’  ([Aichem et al, 2010](#_ENREF_1)) |
| pCMV-HA-UBE2A | Cys88 | AA-390  5’CTGAAGTATGTCCAGAGCTATACTACCATCTGCATAGACATTTGGA 3’  AA-391  5’TCCAAATGTCTATGCAGATGGTAGTATAGCTCTGGACATACTTCAG 3’ |
| pCMV-HA-UBE2B | Cys88 | PR6-21  5`CATCGATTCTGAAGGATATCTAAAGCTATGCTACCATCAGCATACACATT 3`  PR6-22  5`AATGTGTATGCTGATGGTAGCATAGCTTTAGATATCCTTCAGAATCGATG 3` |
| pCMV-HA-UBE2C | Cys114 | AA-380 5’GTGGACACCCAGGGTAACATAGCCCTGGACATCC 3’  AA-381 5’GGATGTCCAGGGCTATGTTACCCTGGGTGTCCAC 3’ |
| pCMV-HA-UBE2D1 | Cys85 | PR6-23  5`GACCTCAGAATATCGAGAGCAATACTTCCATTACTGTTTATGTTTGGATGGT 3`  PR6-24  5`ACCATCCAAACATAAACAGTAATGGAAGTATTGCTCTCGATATTCTGAGGTC 3` |
| pCMV-HA-UBE2D3 | Cys85 | AA-435  5’CATCCAAATATTAACAGTAATGGCAGCATTGCTCTCGATATTCTAAGATCA 3’  AA-436  5’TGATCTTAGAATATCGAGAGCAATGCTGCCATTACTGTTAATATTTGGATG 3’ |
| pCMV-HA-UBE2G2 | Cys89 | AA-392 5’ CCCTGATGGGAGAGTCGCCATTTCCATCCTCCAC 3’  AA-393 5’ GTGGAGGATGGAAATGGCGACTCTCCCATCAGGG 3’ |
| pcDNA5FRT/TO-Strep-HA- UBE2Q2 | Cys304 | AA-405  5’TATGTATTGGGTGGAGGAGCATTAGCTATGGAACTTCTCACAAAAC 3’  AA-406  5’GTTTTGTGAGAAGTTCCATAGCTAATGCTCCTCCACCCAATACATA 3’ |
| pcDNA5FRT/TO-Strep-HA- UBE2QL1 | Cys88 | AA-403 5’ CGGCGGCGCCATCGCCATGGAGCTGCTC 3’  AA-404 5’ GAGCAGCTCCATGGCGATGGCGCCGCCG 3’ |
| pcDNA3.1-3xFLAG-TEV-UBE2O-C1040A | Cys1040 | AA-399 5’ GACAATGGGAAGGTGGCTGTCAGCCTCCTGGG 3’  AA-400 5’ CCCAGGAGGCTGACAGCCACCTTCCCATTGTC 3’ |
| pcDNA3.1-3xFLAG-TEV-  UBE2O-C617A | Cys617 | AA-411  5’GGTACAGTCTGGGGACCACATCGGCCGTACCGCCATGGTGAAGTGGTTCAAGCTGAGGCCGAGTGG 3’  AA-412  5’CCACTCGGCCTCAGCTTGAACCACTTCACCATGGCGGTACGGCCGATGTGGTCCCCAGACTGTACC 3’ |
| pcDNA3.1-3xFLAG-TEV-UBE2O-C617/1040A | Cys617/  Cys1040 | AA-399 5’ GACAATGGGAAGGTGGCTGTCAGCCTCCTGGG 3’  AA-400 5’ CCCAGGAGGCTGACAGCCACCTTCCCATTGTC 3’  AA-411  5’GGTACAGTCTGGGGACCACATCGGCCGTACCGCCATGGTGAAGTGGTTCAAGCTGAGGCCGAGTGG 3’  AA-412  5’CCACTCGGCCTCAGCTTGAACCACTTCACCATGGCGGTACGGCCGATGTGGTCCCCAGACTGTACC 3’ |
