# Supplementary material for: Tumor necrosis factor mediates USE1-independent FAT10ylation under inflammatory conditions
Source: Life Sci Alliance. 2023 Aug 21;6(11):e202301985. doi: 10.26508/lsa.202301985 (PMC10442930; doi:10.26508/lsa.202301985)
Supplement: Supplementary file 4 [file LSA-2023-01985_TableS3.docx]

**Table S3**

| **Plasmid** | **Reference** | **Addgene plasmid** |
| --- | --- | --- |
| pDEST17-UBE2A | ([Jin et al, 2007](#_ENREF_2)) | #15780 |
| pDEST17-UBE2B | ([Jin et al, 2007](#_ENREF_2)) | #15781 |
| pDEST17-UBE2C | ([Jin et al, 2007](#_ENREF_2)) | #15779 |
| pET15-UBE2D1 | ([Jin et al, 2007](#_ENREF_2)) | #15782 |
| pET15-UBE2D2 | ([Jin et al, 2007](#_ENREF_2)) | #15783 |
| pDEST17-UBE2D3 | ([Jin et al, 2007](#_ENREF_2)) | #15784 |
| pDEST17-UBE2D4 | ([Jin et al, 2007](#_ENREF_2)) | #15786 |
| pDEST17-UBE2E1 | ([Jin et al, 2007](#_ENREF_2)) | #15787 |
| pDEST17-UBE2E2 | ([Jin et al, 2007](#_ENREF_2)) | #15788 |
| pDEST17-UBE2E3 | ([Jin et al, 2007](#_ENREF_2)) | #15789 |
| pDEST17-UBE2G1 | ([Jin et al, 2007](#_ENREF_2)) | #15790 |
| pDEST17-UBE2G2 | ([Jin et al, 2007](#_ENREF_2)) | #15791 |
| pDEST17-UBE2T | ([Jin et al, 2007](#_ENREF_2)) | #15808 |
